# Symmetrical electrophysiological brain responses to unilateral and bilateral auditory stimuli suggest disrupted spatial processing in schizophrenia

**DOI:** 10.1038/s41598-019-52931-x

**Published:** 2019-11-11

**Authors:** Sara Sardari, Ali Mohammad Pourrahimi, Hossein Talebi, Shahrzad Mazhari

**Affiliations:** 1 Neuroscience Research center, Institute of Neuropharmacology, Kerman University of Medical Sciences, Kerman, Iran; 2 Department of Psychiatry, Medical School, Kerman University of Medical Sciences, Kerman, Iran; 3 Audiology department, Rehabilitation faculty, Isfahan University of Medical Sciences, Isfahan, Iran

**Keywords:** Cortex, Schizophrenia

## Abstract

Research has found auditory spatial processing deficits in patients with schizophrenia (SCZ), but no study has examined SCZ patients’ auditory spatial processing at both pre-attentional and attentional stages. To address this gap, we investigated schizophrenics’ brain responses to sounds originating from different locations (right, left, and bilateral sources). The event-related potentials (ERPs) of 25 chronic schizophrenic patients and 25 healthy subjects were compared. Mismatch negativity (MMN) in response to frequency and duration deviants was assessed. Two P3 components (P3a and P3b) were elicited via a frequency discrimination task, and MMN and P3 were recorded through separate monaural and dichotic stimulation paradigms. Our results corroborated the previously published finding that MMN, P3a, and P3b amplitudes are reduced in SCZ patients, but they showed no significant effect of stimulus location on either MMN or P3. These results indicated similarity between the SCZ patients and healthy individuals as regards patterns of ERP responses to stimuli that come from different directions. No evidence of auditory hemispatial bias in the SCZ patients was found, supporting the existence of non-lateralized spatial processing deficits in such patients and suggesting compensatory changes in the hemispheric laterality of patients’ brains.

## Introduction

The processing of the spatial location of incoming stimuli is a crucial function of the brain as it enables spatial orientation and coherent perception of the world. Patients with schizophrenia (SCZ) experience a variety of daily life problems, many of which are rooted in spatial processing deficits^1–5^. Studies on spatial-visual processing in SCZ presented strong evidence of right- or left-side bias toward one side of the sensorium, which results in the failure to orient attention to stimuli from one side of space and the consequent loss of stimulus perception^4,6–8^. Hemispatial neglect is an extreme cause of spatial unawareness that is defined as the possibility of completely missing information from a given spatial region. Although this condition is usually observed in patients with right-brain lesions^9^, SCZ individuals have also been suggested to suffer from neglect-like disorders^10^. Recent research further showed that SCZ individuals may detect simultaneously the presented bilateral visual stimuli, but fail to recognize unilateral stimuli, which is another spatial processing disorder called anti-extinction^11^. As can be seen, abundant studies have been devoted to spatial attention and processing in relation to visual modality in SCZ, but little is known about unilateral and bilateral spatial processing in auditory modality. The auditory regions of the cerebral cortex are located in the superior temporal gyrus (STG), which contains the primary auditory cortex (Heschl’s gyrus)^12^. Neuroimaging and post-mortem studies found that SCZ patients exhibit structural changes in auditory regions, particularly the STG^13^, and research on auditory event-related potentials (ERPs) provided strong evidence of cortical processing dysfunctions in SCZ subjects^14,15^. In one ERP investigation, for example, SCZ patients less accurately determined the location of a sound source than did healthy controls^16^.

Given the critical role of spatial processes in everyday living, the present work compared the auditory spatial functioning of SCZ patients and healthy individuals. The main goal was to ascertain whether brain-evoked potentials in response to right- and left-side sounds differ in terms of amplitude and latency within and between study groups. The study was also intended to determine dissimilarities between the groups with respect to ERPs evoked by bilateral sounds. To these ends, we implemented a set of ERP measures to obtain insights into spatial auditory processing in SCZ patients and assessed mismatch negativity (MMN) in response to both frequency and duration deviants. MMN responses reflect an automatic or pre-conscious process of detecting a *mismatch* between frequently and rarely encountered stimuli (e.g., difference in sound duration or pitch)^17^. Because of the subconscious nature of MMN, a logical approach was to examine higher-order cognitive processes—a requirement that we completed by evoking P3a (novelty P3) and P3b ERP components using separate tasks. P3a is elicited via task-irrelevant novel stimuli, whose saliency enable them to capture subject’s attention, even though these triggers are unintentionally heeded^18^. The key difference between P3a and MMN, then, lies in the level of consciousness involved; that is, subtle differences between frequent and deviant stimuli are ignored under MMN, especially if an individual is distracted^19^. P3b is elicited by attended task-relevant stimuli and reflects the allocation of attentional resources when a target stimulus engages memory operations during task performance^20^.

## Results

### Subjects’ characteristics

Table 1 shows the demographic and clinical characteristics of the participants. The healthy controls (HC) and the SCZ patients were matched in terms of age, gender, and educational level. The MMN and P3 amplitudes and latencies were subjected to repeated-measures ANOVA involving one between-group factor (HC, SCZ), two within-group factors of region (Fz, Cz, and Pz electrodes), and stimulus location (left, right, dichotic).

**Table 1:** Demographic and clinical characteristics of the participants (mean ± SD).

Variable	Patients N = 25	Controls N = 25
Education	11.0 ± 3.0	12.0 ± 1.3
Gender N (% male)	22 (88%)	22 (88%)
Edinburgh	95.2 (9.5)	96.5 (9.0)
PANSS Positive	14.0 (4.1)	—
PANSS Negative	16.9 (4.8)	—
Length of the illness (year)	13 (8.6)	—
Chlorpromazine equivalent dose (mg/day)^a^	457	—

^a^Chlorpromazine equivalent dose. Abbreviation: PANSS: positive and negative symptom scale.

### Right versus left stimulus presentation

**Figure 1:**
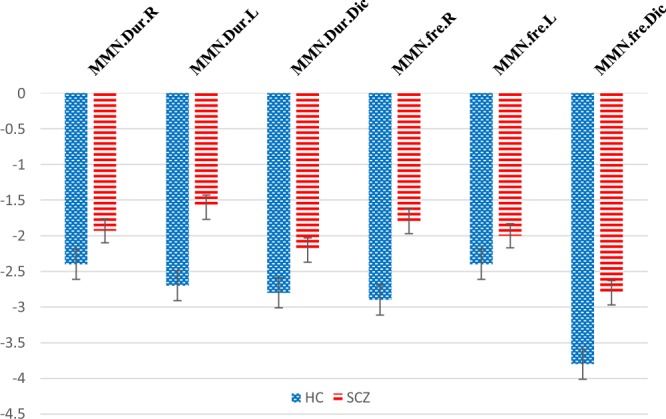
MMN amplitudes in patients (SCZ) and controls (HC).

**Figure 2:**
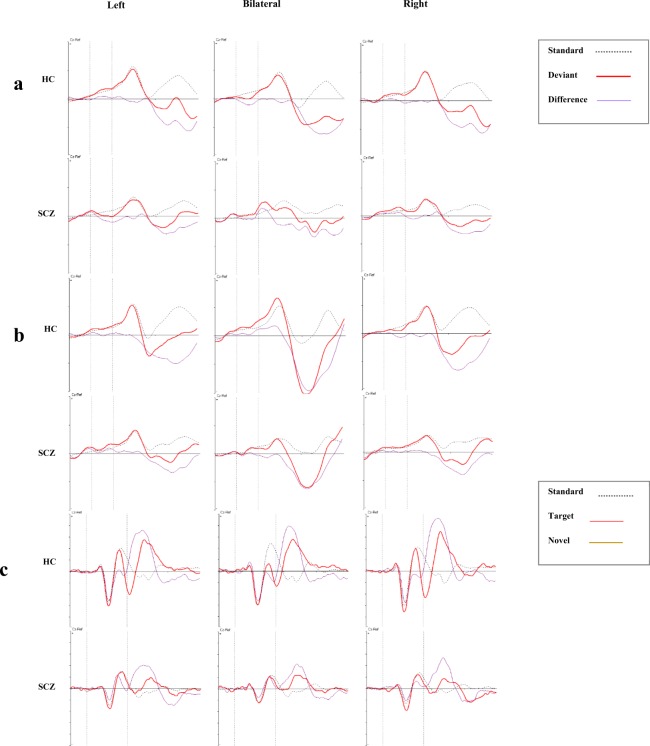
Grand average ERPs at Cz in response to stimuli origination from the left (left column), bilateral (central column), and right (right column) directions (HC: upper row, SCZ: lower row). (**a**) duration MMN, (**b**) frequency MMN, (**c**) P3a and P3b components.

Figure 1 shows the frequency and duration MMN amplitudes in HC and SCZ groups. The duration and frequency MMN grand averages for each group are depicted in Fig. 2a,b, respectively. With regard to duration MMN, ANOVA showed that group composition exerted a significant main effect on amplitude [*F* (1, 38 = 4.6), *p* = 0.02, ŋ^2^ = 0.1] and latency [*F* (1, 38 = 4.1), *p* = 0.04, ŋ^2^ = 0.09] (Fig. 1). Follow-up comparisons showed that the SCZ patients exhibited significantly smaller amplitudes and shorter latencies for both right- and left-side stimuli (*p* < 0.05). Stimulus location had no main effect on amplitude or latency in all directions (*p* > 0.05). With respect to frequency MMN, grouping did not exert a significant main effect on amplitude [*F* (1, 36 = 3.6), *p* = 0.06, ŋ^2^ = 0.09] or latency [*F* (1, 36 = 2.8), *p* = 0.1, ŋ^2^ = 0.07]. As with duration MMN, frequency MMN reflected no significant effect of stimulus location on amplitude or latency (*p* > 0.05).

**Figure 3:**
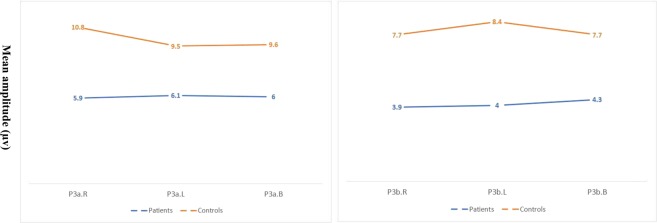
P3a (A) and P3b (B) amplitudes for right (R), left (L), and bilateral dichotic (dic) directions (SCZ vs. HC).

P3a and P3b grand averages for each group are depicted in Fig. 2c. Figure 3 compares the P3a (left) and the P3b (right) amplitudes in the groups for unilateral and bilateral stimulation. The model indicated a significant effect of group composition on P3a amplitudes [*F* (1, 43) = 24.7, *p* < 0.001, ŋ^2^ = 0.3]. A follow-up analysis showed that the SCZ group had significantly smaller amplitudes for left- and right-originating stimuli (*p* < 0.001) but that stimulus location exerted no significant effect [*F* (1, 43) = 1.2, *p* = 0.2, ŋ^2^ = 0.02]. The model also showed that group composition caused a significant main effect on P3b amplitudes [*F* (1, 43) = 23.7, *p* < 0.001, ŋ^2^ = 0.3]. The SCZ patients had significantly smaller amplitudes for stimuli originating from both left and right (*p* < 0.001). Stimulus location did not cause any significant main effect on P3b amplitudes [*F* (1, 43) = 1.5, *p* = 0. 2, ŋ^2^ = 0.03]. The group composition effects on P3a and P3b latencies were also nonsignificant (*p* > 0.05). Table 2 presents a detailed description of ERP amplitudes and latencies in HCs and SCZs.

**Table 2:** ERP mean amplitude and latency of HCs and SCZ patients in electrode regions [M ± SD (SE)].

ERP component	Deviance type	Direction	Right						Left						Both					
		Region	Fz		Cz		Pz		Fz		Cz		Pz		Fz		Cz		Pz	
		group	Amp.	Lat.	Amp.	Lat.	Amp.	Lat.	Amp.	Lat.	Amp.	Lat.	Amp.	Lat.	Amp.	Lat.	Amp.	Lat.	Amp.	Lat.
**MMN**	Duration	HC	−2.6 ± 1.1 (0.2)	204.3 ± 29.3 (6.1)	−2.9 ± 1.3 (0.2)	199.9 ± 30.3 (6.3)	−1.8 ± 1.2 (0.2)	201.3 ± 30.7 (6.4)	−3 ± 1.5 (0.3)	203.2 ± 29.5 (6)	−3 ± 1.4 (0.2)	204.3 ± 27 (5.5)	−2.2 ± 0.9 (0.1)	199.7 ± 28.2 (5.7)	−3.1 ± 1.8 (0.3)	206.1 ± 27.1 (5.5)	−3.2 ± 1.5 (0.3)	204.5 ± 23.9 (4.8)	−2.3 ± 1 (0.2)	202.6 ± 25.4 (5.1)
		SCZ	−2 ± 10 (0.2)	190.8 ± 35.9 (8.2)	−2 ± 1 (0.2)	181.4 ± 39.1 (8.7)	−1.8 ± 1.2 (0.2)	186.8 ± 25.5 (5.8)	−2 ± 1.3 (0.3)	185 ± 27.1 (6.0)	−1.9 ± 1.1 (0.2)	186.1 ± 29.5 (6.2)	−1.1 ± 1 (0.2)	185.3 ± 25.9 (5.8)	−2.1 ± 1.3 (0.3)	186.8 ± 32.6 (7.4)	−2.2 ± 1.7 (0.3)	182.3 ± 40.7 (8.9)	−2.4 ± 2 (0.5)	174.1 ± 34.9 (8.0)
	Frequency	HC	−3.1 ± 1.5 (0.3)	183.7 ± 27 (5.6)	−3.2 ± 1.8 (0.3)	180.6 ± 2.7 (4.9)	−2.4 ± 1.3 (0.2)	184.9 ± 31.3 (6.5)	−2.9 ± 1.3 (0.2)	202 ± 29 (6)	−2.6 ± 1.3 (0.2)	188.2 ± 34 (7)	−1.8 ± 1 (0.2)	176.9 ± 38.5 (8)	−4.4 ± 1.4 (0.3)	172.7 ± 17.6 (3.6)	−4.1 ± 1.5 (0.3)	174.8 ± 22 (4.5)	−2.9 ± 1.2 (0.2)	176 ± 24.3 (5)
		SCZ	−2.1 ± 1.6 (0.4)	178.4 ± 26.3 (6.8)	−1.8 ± 1.5 (0.3)	169.6 ± 26.5 (6.6)	−1.5 ± 1.2 (0.3)	159.2 ± 26.2 (6.7)	−2.3 ± 1 (0.2)	186.6 ± 34.7 (8.6)	−2.1 ± 1.4 (0.3)	182.2 ± 288 (7.0)	−1.7 ± 1.5 (0.3)	172.6 ± 36.5 (9.1)	−3.5 ± 1.4 (0.3)	174.1 ± 24.8 (6)	−3 ± 1.1 (0.2)	171.3 ± 20.9 (4.9)	−2.1 ± 0.8 (0.2)	166.5 ± 26.5 (6.4)
**P3a**		HC	10.7 ± 3.8 (0.7)	340.1 ± 43.2 (8.8)	12.0 ± 4.1 (0.8)	323 ± 41.7 (8.5)	9.8 ± 3.6 (0.7)	346.5 ± 37.6 (7.6)	9.2 ± 4.0 (0.8)	335.1 ± 45.2 (9.4)	10.3 ± 4.3 (0.9)	333.7 ± 41.1 (8.5)	9.0 ± 3.2 (0.6)	342 ± 45.9 (9.5)	9.6 ± 5.1 (1)	343.3 ± 32.4 (6.7)	10.7 ± 5 (1)	334.1 ± 34.5 (7.1)	8.5 ± 3.5 (0.7)	356.6 ± 43 (8.9)
		SCZ	5.6 ± 2.9	353.9 ± 53.8	6.5 ± 3.3	343.1 ± 56.2	5.9 ± 2.9	363.1 ± 57	5.7 ± 2.8 (0.5)	357.1 ± 64.3 (13.4)	6.8 ± 3.5 (0.7)	355.8 ± 53.7 (11.1)	5.9 ± 3.2 (0.6)	374.6 ± 46.7 (9.7)	5.8 ± 3.4 (0.6)	357.6 ± 63.9 (13)	6.7 ± 3.2 (0.6)	356 ± 64.5 (13.1)	5.7 ± 2.4 (0.5)	371.9 ± 63.9 (13)
**P3b**		Region	Pz		P3		P4		Pz		P3		P4		Pz		P3		P4	
		HC	8.4 ± 3.8 (0.7)	384.3 ± 54.4 (11.1)	7.4 ± 3.7 (0.7)	388.2 ± 55.7 (11.3)	7.4 ± 3.5 (0.7)	385.1 ± 58.5 (11.9)	9.1 ± 3.8 (0.8)	387.8 ± 53.1 (11.0)	8 ± 3.8 (0.8)	390.2 ± 44.9 (9.3)	8.2 ± 3.3 (0.6)	393.0 ± 51.7 (10.7)	8.4 ± 3.5 (0.7)	377 ± 67.4 (14)	7.1 ± 3.2 (0.6)	396 ± 62.3 (12.9)	7.7 ± 3.5 (0.7)	374 ± 68.2 (14.2)
		SCZ	4.4 ± 2.3 (0.4)	416.4 ± 80.7 (16.8)	3.8 ± 1.7 (0.3)	410.6 ± 74.7 (15.5)	3.7 ± 1.8 (0.3)	401.8 ± 89 (18.5)	4.3 ± 2.6 (0.5)	432.5 ± 85.7 (17.8)	3.6 ± 2.6 (0.5)	409 ± 79.4 (16.5)	4.1 ± 2.3 (0.4)	419.8 ± 71.4 (14.8)	4.4 ± 2.4 (0.5)	451.8 ± 85.8 (17.5)	3.8 ± 2.3 (0.4)	450.5 ± 85.4 (17.4)	4.3 ± 1.7 (0.3)	444.4 ± 90.1 (18.3)

### Binaural versus unilateral stimulus presentation

Concerning duration MMN, the group composition exerted a significant main effect on amplitude [*F* (1, 38) = 4.2, *p* = 0.04, ŋ^2^ = 0.1] and latency [*F* (1, 38) = 6.9, *p* = 0.01, ŋ^2^ = 0.1]. This finding is attributed to the significantly smaller amplitudes and shorter latencies of the SCZ patients at all stimulus directions (*p* < 0.05). Stimulus location had a nonsignificant effect on amplitude and latency (*p* < 0.05). As to frequency MMN, the group composition exerted a significant main effect on amplitude [*F* (1,36) = 4.6, *p* = 0.03, ŋ^2^ = 0.1), and the SCZ patients had significantly smaller amplitudes than did the controls for all stimulus locations (*p* < 0.05). Stimulus location exerted a significant effect [*F* (2, 72) = 20.0, *p* < 0.001, ŋ^2^ = 0.3], indicating changes in amplitude at different stimulus directions. The interaction between stimulus direction and group composition was nonsignificant [*F* (2, 72) = 1.2, *p* = 0.2, ŋ^2^ = 0.03], suggesting that the rates of amplitude variations were similar across the groups. The model indicated that group composition exerted no significant main effect on latency [*F* (1, 36) = 2.6, *p* = 0.1, ŋ^2^ = 0.06] but that stimulus direction significantly affected amplitude [*F* (2, 72) = 4.5, *p* = 0.01, ŋ^2^ = 0.1]. These results are ascribed to the significantly shorter latencies among the SCZ patients than among the HCs for left and bilateral sources of stimuli (*p* < 0.05). The interaction between stimulus direction and grouping was nonsignificant [*F* (2, 72) = 0.4, *p* = 0.4, ŋ^2^ = 0.01], implying that the patterns of amplitude variations were similar across the groups.

In connection with P3a, the statistical model showed a significant main effect of group composition on amplitudes [*F* (1, 42) = 22.3, *p* < 0.001, ŋ^2^ = 0.3] and latencies [*F* (1, 42) = 6.0), *p* = 0.01, ŋ^2^ = 0.1]. The P3a amplitudes were significantly smaller, and the latencies were significantly longer among the SCZ patients in all stimulus directions (all *p* < 0.05). Stimulus direction did not significantly influence amplitude [*F* (2, 84) = 0.7, *p* < 0.4, ŋ^2^ = 0.01] or latency [*F* (2, 84) = 0.7), *p* = 0.4, ŋ^2^ = 0.01], suggesting similarity in amplitude changes with directional variations. In relation to P3b, the model showed a main effect of group composition on amplitudes [*F* (1, 42) = 23.7), *p* < 0.001), ŋ^2^ = 0.3] and latencies [*F* (1, 42) = 6.5), *p* = 0.01), ŋ^2^ = 0.1]. This finding is due to the significantly smaller P3b amplitudes and longer latencies among the SCZ patients for all stimulus locations (*p* > 0.5). Stimulus location did not significantly affect P3b amplitude or latency.

## Discussion

This study investigated the ERPs evoked by unilateral and bilateral sounds at different attentional levels, namely, (1) subconscious attention to subtle differences in sensory inputs (MMN), (2) conscious but unwanted attention to attention-capturing stimuli (P3a), and (3) voluntary and goal-directed attention (P3b) in SCZ. Consistent with imaging^21^ and ERP^22^ studies, our results demonstrated overall reduced MMN, P3a, and P3b amplitudes in the SCZ patients, indicating that the trials were sensitive to the differentiation of the patients from the controls. However, the effects of stimulus location on the ERP amplitudes and latencies of the groups were comparable. We discuss this finding for the unilateral and bilateral stimulus presentations separately.

In case of right vs left side stimulation, the symmetric MMN responses to different locations of frequency or pitch deviance in the SCZ patients suggested a dysfunction in the automatic comparison of an existing memory trace with incoming novel stimuli, which were unaffected by stimulus direction. There are contradictory reports on the patterns of MMN responses to lateralized stimuli in SCZ. Our findings support those of previous studies in that they implied a symmetric volume loss or hypoactivation of the STG in SCZ^21,23,24^. As discussed in past research, because the STG is the main brain-related generator of MMN, its anomalies can result in the well-known MMN amplitude decrement in schizophrenic patients. Post-mortem studies on SCZ patients also showed neuronal loss in auditory-related areas of the anterior cingulate cortex (ACC)^25,26^ and mid-dorsolateral prefrontal cortices^27^. With regard to robust anatomical links between ACC and the frontal auditory areas^27^, the descent of inputs from the latter to the former can induce a symmetric drop in ERP responses^28,29^, as was observed in the present work.

Similar to the MMN, the P3a and P3b components indicated that stimulus direction exerted comparable effects on the amplitudes and latencies of the two groups—a phenomenon that can be related to a general deficit in both hemispheres. These findings are consistent with some research that reported the bilateral hypoactivation of P3a sources^30–32^ and P3b generators^32–34^.

When comparing unilateral vs bilateral stimulation, our results revealed comparable responses to binaural and unilateral auditory stimuli. The P3a and P3b deficits observed in the present work and similar studies uncovered that target and distractor processing involves distinct subsystems and that such processing types are altered in SCZ in a symmetrical pattern^35^. Consistent with our findings, imaging studies reported frontal lobe and cingulate hypofunction in SCZ; that is, the frontal lobe engages in discriminatory tasks, which is essential for P3 generation^36^, whereas the cingulate gyrus is involved in the effortful initiation (P3b) and inhibition (P3a) of motor responses^37^. On a battery of psychological tasks, SCZ patients showed significant impairments in tests that were sensitive to right or left frontal and temporal lobe damage, but they performed normally in tests that were sensitive to parietal lobe lesions^38^. The frontal and temporal lobes may thus be bilaterally involved in SCZ. In contrast to research reports of right-^15^ or leftward^39^ bias in the sensorium, some findings pointed to a lack of asymmetry in MMN^40^ or P3^16,20,41^ responses to dichotic stimuli. These inconsistent results may have stemmed from differences in patient samples among different laboratories^42–44^. Simple ERP tasks may not effectively uncover asymmetric language-related dysfunctions in SCZ, thus highlighting the need for more complex and demanding tasks containing more phonological content^20,45^.

Switching attention to a change in an incoming signal is accomplished through a set of responses: N1 as the index of transient detection, MMN for sensory-memory-based change detection, P3a for involuntary attention orientation, and eventually, the task-related voluntary response or P3b^46–48^. Although dissociated in mechanisms^20,35,49^, these brain waves play complementary roles in the aforementioned shift in attention^48^. The symmetric deficits of ERP responses to different sound directions in the present work could be an indicator of pervasive deficits in change detection systems, which are responsible for both bottom–up (MMN) and top–down (P3) processes^50^. Nevertheless, this pattern of findings does not mean that spatial processing abnormalities are nonexistent in SCZ patients. Such pattern can be interpreted as being related to some nonlateralized dysfunctions in spatial processing.

Two perspectives have been proposed to explain spatial attention bias: the first is the well-known concept of lateralized deficits, which suggest that brain dysfunction in one hemisphere affects the contralesional hemispace^51,52^, and the second is the issue of non-lateralized impairments, at which the spatial deficits might not necessarily worsen toward one side of a space^13^. Researchers have also argued that such a whole space deficit can be related to the ACC hypofunction in SCZ^28,29^.

Interestingly, spatial processing deficits may involve not only attending to stimuli but also ignoring stimuli from different locations. As demonstrated in a previous study, SCZ patients failed to filter peripheral (right vs. left) stimuli when they were required to focus centrally^1^. In line with our findings, the researchers reported no difference between HCs and SCZ patients in ignoring outer peripheral stimuli. They attributed this observation to a hyperfocusing of attention to central locations of the sensorium in SCZ individuals, along with a disability to distribute attention broadly to the periphery.

Another potential cause of the symmetric pattern of brain responses in the current study are plastic changes in the brain during the course of SCZ^48^. Hypoactivation of the left hemisphere, including the left temporal lobe, is a frequent occurrence in schizophrenic patients^53,54^, and this condition can progress, be altered, or be reversed during the course of the disease^55^. Therefore, temporal lobe functions, including spatial coding, may alter during the course of disease. As a compensatory reaction, the brain may decrease the right STG activation with the course of the disease; hence, a symmetrical hypoactivation takes place, as was observed in the present work.

To the best of our knowledge, this research is the first to use a dichotic listening paradigm in examining the spatial correlates of auditory attention among SCZ patients and assess auditory spatial functions at multiple levels of consciousness. On the basis of the sequential model of auditory perception^56^, the perceptual segregation and spatial localization of auditory objects are the prerequisites of higher-order auditory processes, including dichotic listening paradigms^57^. Given the sensitivity of dichotic listening tasks to auditory stream segregation and fusion, they can serve as a valuable tool for spatial processing in ERP studies^58–60^. Another methodological strength of the present work was the sound field testing, which more closely approximates real-world situations than does tests involving ear covering. An ideal improvement to this approach would be to conceal sound sources to eliminate visual cues.

The limitations of this study are worth noting. First, all the patients were taking their medications, which might have affected the results. Future studies should examine auditory processing across a group of drug-naive patients and patients with different subtypes of SCZ. Second, the patient group was recruited mainly from an outpatient clinic and showed mild symptoms. Thus, they may not exactly represent individuals typically encountered in clinical practice or examined in previous SCZ studies, thereby potentially limiting the generalizability of the findings. Note that it would be helpful if patients at different stages of SCZ (for example, in the groups of first episode and chronic patients, or in the form of longitudinal surveys) be involved in such evaluations, which helps the researchers to study changes during the disease. Concerning the potential effect of the medicines on the disease course, similar research may help to understand their long-lasting effects on the patients’ functions, as an invaluable source for promoting the policies of medical therapy in SCZ patients. Moreover, specifying the characteristics and pattern of spatial processing deficits in SCZ helps the therapists in developing beneficial cognitive therapies for these patients. For example, if the SCZ patients underestimated the peripheral (right and left) stimuli, but focused intensely on the stimuli from the midline of the sensorium, it might be possible to suggest a rehabilitative plan to balance this abnormal spatial function.

Conclusively, using pre-attentional and attention-dependent measures, we found deficits in detecting, orienting, and responding to changes in auditory sensory inputs among the SCZ patients. This phenomenon is most probably related to a general hypofunction rather than a lateralized bias in response to different stimulus directions.

## Methods

### Participants

Thirty-five right-handed outpatient schizophrenic patients whose characteristics corresponded with the DSM IV (Diagnostic and Statistical Manual of Mental Disorders, fourth edition) criteria for SCZ were assessed using the positive and negative symptom scale^61^. The patients did not have a history of electroconvulsive therapy within six months prior to the testing period. They were receiving their medications at the time of testing at a mean chlorpromazine equivalent dose of 457 mg/Kg.

The control group was composed of 35 right-handed healthy individuals who were screened for personal and family histories of psychotic illnesses. The exclusion criteria were a history of neurological disorders and head injury as well as current substance abuse. All the participants were assessed via behavioral audiometry (based on the modified Hughson–Westlake procedure) configured to four frequency octave bands (0.5, 1, 2, and 4 KHz). Normal hearing was defined as hearing thresholds ≤25 dB. Each subject was paid US $ 20 participation fee.

The study was conducted under the principles of the Declaration of Helsinki for Biomedical Research. Accordingly, written informed consent was obtained from all the participants, and the study was approved by the Ethics Committee of Kerman University of Medical Sciences.

### Assessment procedures

All the experiments were performed in a sound-proof, dimly lit chamber with ambient noise levels that satisfied the ANSI S3.1-1999 standards for audiometry chambers. The sound stimuli included pure tones, which were presented from one or two speakers located at the ear level (distance from the head center: 90 cm, hearing angle: ±90° azimuth) and covered with suitable curtains. The participants were seated comfortably, with their head fixed in a relaxed posture on a chin rest. The sequence of tasks, which were designed using PsyTask version 1.53.17 (Mitsar Inc., Russia), was counterbalanced across the participants.

### MMN recording

The participants were instructed to silently watch a wildlife movie on a monitor (distance from eyes: 80 cm, visual angle: 5° on each side from the mid-sagittal plane) and disregard sounds. Each block consisted of 1500 stimuli, including 150 rare (deviant) and 1350 frequent (standard) tones (deviance probability: 10%). Each trial was characterized by 50 ms of pre-stimulus time, 50 ms (or 100 ms for duration deviants) of stimulus presentation, 150 ms of interstimulus intervals (ISIs), and a sequential interdeviant interval of 9 (with each successive deviant disrupted by 5 to 13 standards). Regarding the previous reports of different response patterns to frequency vs duration deviance in SCZ studies^62^, both paradigms were implemented in the present work. With respect to duration MMN, the unilateral stimuli were tones with a frequency of 600 Hz, each presented for 50 ms (as standard stimuli) and 100 ms (as deviants), once from the right side and once again from the left. Bilateral stimuli were a pair of tones, once with a frequency of 740 Hz that were always presented from the left and another tone with a frequency of 932 Hz that was always presented from the right. These tones were presented for 50 ms (as standard stimuli) and 100 ms (as deviant stimuli). The tasks were designed with consideration of the works of Deouell *et al*.^63^ and Kayser *et al*.^20^.

With regard to frequency MMN, the unilateral stimuli were 485 Hz tones as the standards and 444 Hz as the deviants (with each pair presented once from the right and once from the left). In dichotic stimulation, a 740 Hz tone from the left and a 932 Hz tone from the right were presented as standard stimuli, and a 660 Hz tone from the left and a 830 Hz tone from the right were presented as deviant stimuli, all at a duration of 50 ms. All the bilateral (dichotic) stimuli were 6 dB weaker in intensity compared with the unilateral stimuli.

### P3 recording

A three-stimulus oddball paradigm was applied in P3 elicitation. Each block consisted of 600 stimuli, namely, 60 targets (probability: 5%), 60 familiar stimuli, environmental novels^64,65^ (probability: 5%), and 480 standard tones. Each trial was characterized by 100 ms of pre-stimulus time, 250 ms of stimulus duration, and 1 s of ISI. Every successive target was disrupted by 2 to 5 standards. In the unilateral tests, a 1 KHz tone was used as a standard stimulus, and a 1.5 KHz tone was employed as the target (each presented once from the right and once from the left). In the dichotic tests, a 444 Hz tone from the left combined with a 485 Hz tone from the right was adopted as a standard stimulus, and a 1084 Hz tone originating from the left paired with a 1125 Hz tone coming from the right was used (the novel sound was also played dichotically). This noticeable frequency difference between standard and deviant stimuli in P3 task (compared with the MMN recording paradigm) was considered according to previous reports of frequency discrimination problems in some SCZ patients^66^. The participants were instructed to ignore the novel sounds and press the space bar of a keyboard when they hear a high-pitched tone.

### ERP acquisition

Electroencephalography (EEG) data were recorded and analyzed using a 32-channel Win EEG System (version 2.126.97, Mitsar Inc., Russia), with a sampling rate of 250 Hz, a low-pass filter off 0.1 Hz, a high-pass filter of 45 Hz, and band notch of 45 to 55 Hz. Electrode impedances were kept below 5 kΩ.

The raw EEG was recorded to the database using monopolar montage in relation to reference electrodes (average reference). The EEG was then reformatted and filtered according to the montage parameters (it can be modified by the command: Setup > Montage List) into the selected montage (monopolar 1 A1 < - > A2), and the data were re-referenced offline to linked ear lobes.

Motion correction was performed in 4 steps: 1. Visual monitoring of the raw EEG and removing the noisy trials manually. The noisy trials were selected based on their very large amplitude (for muscle artifacts, usually over 100 µv) and their morphology. 2. Removing the eye blinks (in the Win EEG software it can be done by de- selecting the Bio channel in the “change montage” tab). 3. Selecting a clean, well recorded piece of EEG (about 180 trials), as a sample. 4. Running independent component analysis (ICA) through this command: Analyze tab > Artifacts correction. In case of noisy channels, as we monitored the EEG recording online, it was possible to detect the presence of any constant noise in a special channel. In this case, the recording was paused and the noise source (usually, the myogenic activity caused by frowning) was amended. Hence, we had not any “bad channel” in our records to be removed.

The minimum number of clean trials needed for EEG analysis were 70% of all of testing trials (in case of P3 recordings: 490 trials, and for MMN recordings: 735 trials). However, we were usually more cautious that this, as we did not reject more than 20% of trials.

After artifact rejection, ERPs were averaged relative to stimulus onset, which covered 100 ms of pre-stimulus time to 700 ms of post-stimulus. MMN was identified as the largest negative valley in the latency range of 110 to 250 ms at mid-sagittal electrodes (Fz, Cz, and Pz). P3a and P3b components were defined as the largest peaks in the latency range of 230 to 450 ms and 300 to 600 ms after stimulus onset, respectively. Fz, F3, F4, Cz, and Pz electrodes were selected for P3a analysis, and parietal electrodes (P3, Pz, and P4) were selected for P3b analysis.

### Statistical analyses

A repeated measure analysis of variance (ANOVA) was conducted to test for the effects of two within subjects’ factor: 1. The stimulus location (three levels: unilateral right, unilateral left and bilateral stimulation) and 2. The electrode site (three levels, as described above). Significant main effects, interactions, and follow-up pairwise comparisons were examined after adjustment for multiple testing (Bonferroni). A combination of chi-square tests and t-tests were used on demographic and clinical data. All the analyses were conducted using the Statistical Package for the Social Sciences version 20.

The sample size was determined based on similar previous studies^15,20^. The power analysis was performed using the G*power software to determine the number of subjects that would have been needed to indicate the difference in amplitude and latencies (α = 5% and power = 80%).

## Data Availability

The datasets generated and/or analyzed during the study are available from the corresponding author upon request.
